# Rescue therapy with Tanshinone IIA hinders transition of acute kidney injury to chronic kidney disease *via* targeting GSK3β

**DOI:** 10.1038/srep36698

**Published:** 2016-11-18

**Authors:** Chunming Jiang, Wei Zhu, Xiang Yan, Qiuyuan Shao, Biao Xu, Miao Zhang, Rujun Gong

**Affiliations:** 1 Department of Nephrology, Affiliated Nanjing Drum Tower Hospital, Nanjing University School of Medicine, Nanjing, China; 2 Division of Kidney Disease and Hypertension, Department of Medicine, Rhode Island Hospital, Brown University School of Medicine, Providence, Rhode Island, USA; 3 Department of Urology, Affiliated Nanjing Drum Tower Hospital, Nanjing University School of Medicine, Nanjing, China; 4 Department of Cardiology, Affiliated Nanjing Drum Tower Hospital, Nanjing University School of Medicine, Nanjing, China

## Abstract

Acute kidney injury (AKI) remains challenging for clinical practice and poses a risk of developing progressive chronic kidney disease (CKD) with no definitive treatment available yet. Tanshinone IIA, an active ingredient of Chinese herbal *Salvia miltiorrhiza*, has been widely used in Asia for the remarkable organoprotective activities. Its effect on established AKI, however, remains unknown. In mice with folic acid-induced AKI, delayed treatment with Tanshinone IIA, commenced early or late after injury, diminished renal expression of kidney injury markers, reduced apoptosis and improved kidney dysfunction, concomitant with mitigated histologic signs of AKI to CKD transition, including interstitial fibrosis and tubular atrophy, and with an ameliorated inflammatory infiltration in tubulointerstitium and a favored M2-skewed macrophage polarization. Mechanistically, Tanshinone IIA blunted glycogen synthase kinase (GSK)3β overactivity and hyperactivation of its downstream mitogen-activated protein kinases that are centrally implicated in renal fibrogenesis and inflammation. Inhibition of GSK3β is likely a key mechanism mediating the therapeutic activity of Tanshinone IIA, because sodium nitroprusside, a GSK3β activator, largely offset its renoprotective effect. In confirmatory studies, rescue treatment with Tanshinone IIA likewise ameliorated ischemia/reperfusion-induced kidney destruction in mice. Our data suggest that Tanshinone IIA represents a valuable treatment that improves post-AKI kidney salvage *via* targeting GSK3β.

Acute kidney injury (AKI), characterized by an abrupt decline in kidney function, is a common and devastating problem in clinical practice. Epidemiologic evidence indicates a growing prevalence of acute kidney disease worldwide, probably associated with increasing incidence of other comorbidities, including diabetes mellitus, hypertension, and caloric nutritional overload^1^,^2^. Despite enormous efforts dedicated to the understanding of the underlying pathogenic mechanisms, AKI continues to be a therapeutic challenge with no definitive and specific treatment available yet to rescue kidney injury after AKI. Existing regimens are virtually confined to symptomatic treatments and general supportive cares^3^, which are, however, of limited utility with unsatisfying therapeutic efficacy, as evidenced by the poor prognosis. Indeed, long-term follow-up studies show that the number of patients with incomplete renal recovery have been underestimated and that AKI *per se* is an independent risk factor for subsequent transition to chronic kidney disease (CKD) and progression to end stage renal disease^4^,^5^,^6^,^7^,^8^. There is therefore a pressing need to develop novel approaches to promote kidney salvage following AKI. To this end, numerous pre-clinical studies have been carried out in the past several decades. However, the majority of these studies were performed in a preventive manner with the intervention imposed prior to kidney insults, thus making these studies clinically irrelevant^9^,^10^,^11^,^12^. Moreover, as a complex disease, AKI does not have a single cause, but instead involves multiple pathogenic mechanisms^13^, such as renal tubular damage, inflammation, and fibrogenesis. Consequently, most novel approaches targeting merely a single pathogenic pathway eventually failed in late-stage trials even though they exhibited some promise at early phases. It is imperative to explore multi-pronged approaches for kidney rescue after AKI.

Chinese herbal medicine has being practiced for thousands of years and successfully treated a number of human diseases, including some kidney diseases^14^,^15^,^16^. Although the exact pharmacologic mechanisms remain to be clarified, striking advances have been made in recent years by applying modern pharmacochemistry techniques and methods. Tanshinone IIA, an active ingredient of the traditional Chinese herbal *Salvia miltiorrhiza*, has been widely used in Asia to treat various diseases for its remarkable organoprotective activities^14^,^17^,^18^,^19^,^20^,^21^,^22^. Tanshinone IIA has been proven to be a low toxic and safe agent by numerous clinical and experimental studies^19^,^20^,^21^,^22^. Emerging data suggest that Tanshinone IIA possesses significant anti-inflammatory activities, by which it ameliorates injury in various organ systems, such as the brain, lung and heart^23^,^24^,^25^,^26^,^27^,^28^,^29^. Notably, two randomized clinical trials are being undertaken to validate its effect on ischemic heart disease^30^,^31^. In addition, a prominent beneficial effect of Tanshinone IIA in experimental chronic kidney diseases has also been reported, including remnant kidney disease, diabetic nephropathy, uric acid nephropathy, and hypothermic kidney preservation^32^,^33^,^34^,^35^,^36^,^37^. However, the effect of Tanshinone IIA on AKI has been barely examined. This study aimed to determine the effect of delayed Tanshinone IIA treatment on established AKI in murine models of AKI induced by folic acid or ischemia-reperfusion injury (IRI). We found that Tanshinone IIA therapy initiated at either early or late after injury, was effective in protecting against both acute and chronic kidney damage and dysfunction.

## Results

### Delayed Tanshinone IIA treatment improves kidney dysfunction incited by folic acid

**Figure 1:**
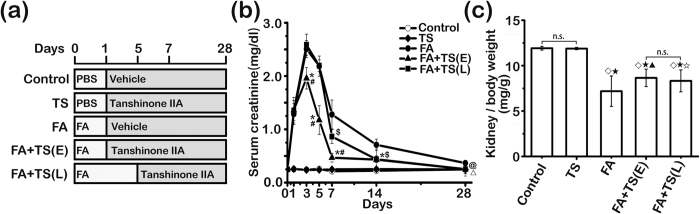
Tanshinone IIA ameliorates folic acid-induced renal dysfunction in mice. (**a**) Animal experimental design. Mice were randomized into 5 groups to receive vehicle, folic acid (FA) and/or Tanshinone IIA (TS) given on day 1 [FA+TS(E)] or day 5 [FA+TS(L)] after folic acid injury; (**b**) Time course changes of serum creatinine levels (mg/dl) measured on days 0, 1, 3, 5, 7, 14 and 28; ^*^P < 0.001, FA+TS(E) *vs* FA; ^#^*P* < 0.001 FA+TS(E) *vs* FA+TS(L); ^$^*P* < 0.001, FA+TS(L) *vs* FA; ^△^*P* = 0.017, FA+TS(E) *vs* FA; ^@^*P* = 0.018, FA+TS(L) *vs* FA; (n = 9). (**c**) Kidney to body weight ratios were determined on day 28. ^◊^*P* < 0.001 *vs* Control; ^★^*P* < 0.001 *vs* TS; ^▲^*P* = 0.004 *vs* FA; ^☆^*P* = 0.024 *vs* FA; n.s., not significant; (n = 9).

Following folic acid injury (Fig. 1a), mice rapidly developed AKI, marked by an elevated serum creatinine level that was evident on day 1 and peaked on day 3. This was followed by a partial spontaneous recovery with the serum creatinine level higher than the normal level till day 28 (Fig. 1b). In contrast, Tanshinone IIA therapy, starting 1 day or 5 days after folic acid injury, significantly attenuated the elevation of serum creatinine levels, and completely corrected kidney dysfunction on day 28. In consistency, gross parenchymal changes in the kidney, reflected by reduced kidney to body weight ratios, were evidently observed 28 days after folic acid injection. This effect was considerably abrogated by Tanshinone IIA treatment with a more prominent efficacy exhibited in the early-treated than the late-treated animals (Fig. 1c).

### Tanshinone IIA reduces kidney injury markers and ameliorates inflammatory infiltration following folic acid insult

**Figure 2:**
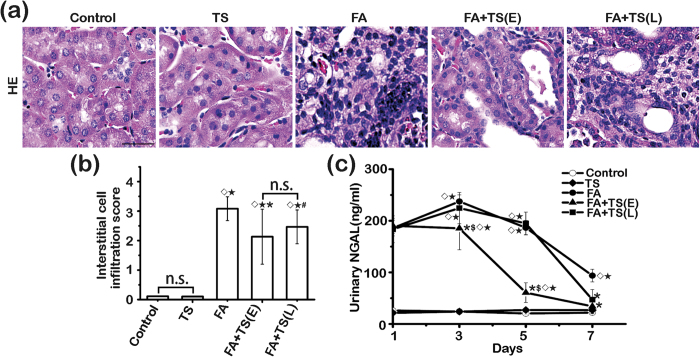
Both early and late Tanshinone IIA treatments ameliorate kidney inflammation and injury induced by folic acid. (**a**) Representative micrographs showing hematoxylin-eosin (HE) staining of kidney specimens procured on day 7. Scale bar = 100 μm. (**b**) Interstitial cell infiltration score assessed based on evaluation of HE staining. ^◊^*P* < 0.001 *vs* Control; ^★^*P* < 0.001 *vs* TS; ^*^P < 0.001 *vs* FA; ^#^*P* = 0.016 *vs* FA; n.s., not significant (n = 9). (**c**) Time course changes of urinary neutrophil gelatinase associated lipocalin (NGAL) levels. ^◊^*P* < 0.001 *vs* Control; ^★^*P* < 0.001 *vs* TS; ^*^P < 0.001 *vs* FA; ^$^*P* < 0.001 *vs* TS + TS(L); n.s., not significant; (n = 9).

To determine the protective effects of Tanshinone IIA on folic acid injured kidney, kidney sections and urine sample were collected for assessment at 7 days after folic acid injection. Seven days after folic acid injection, mouse kidney demonstrated prominent hypercellularity in the interstitium as revealed by hematoxylin and eosin (H&E) staining (Fig. 2a). Tanshinone IIA treatment markedly attenuated renal inflammatory infiltration with a greater efficacy conferred by the early than the late Tanshinone IIA treatment according to scoring of renal interstitial cell infiltration (Fig. 2b). In parallel, the augmented urine excretion of neutrophil gelatinase-associated lipocalin (NGAL) after folic acid injection was diminished in Tanshinone IIA treated mice with a stronger effect observed in the early treatment group (Fig. 2c). Collectively, these findings suggest that Tanshinone IIA possesses an anti-inflammatory property that prevents the progression of established AKI.

### Tanshinone IIA hinders transition of AKI to CKD after folic acid injury

**Figure 3:**
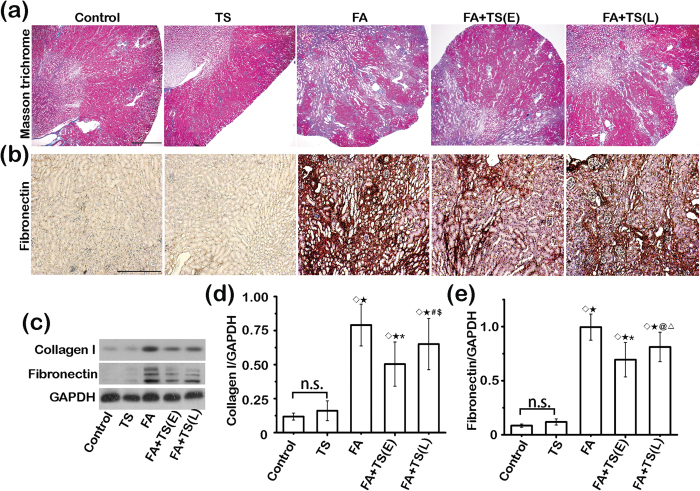
Tanshinone IIA attenuates folic acid-induced kidney fibrosis. (**a**) Representative micrographs showing Masson trichrome staining of kidney specimens procured on day 28; Scale bar = 1 mm. (**b**) Representative micrographs showing immunoperoxidase staining of kidney specimens procured on day 28 for fibronectin; Scale bar = 500 μm. (**c**) Representative immunoblots of collagen I and fibronectin in cortical kidney lysates. (**d**,**e**) Relative abundance of collagen I (**d**) and fibronectin (**e**) in immunoblots expressed as densitometric ratios of collagen I/GAPDH or fibronectin/GAPDH. ^◊^*P* < 0.001 *vs* Control; ^★^*P* < 0.001 *vs* TS; ^*^P < 0.001 *vs* FA; ^#^*P* = 0.034 *vs* FA; ^$^*P* = 0.026 *vs* FA+TS(E); ^@^*P* = 0.001 *vs* FA; ^△^*P* = 0.027 *vs* FA+TS(E); n.s., not significant; (n = 9).

To determine the exact histologic changes in the kidney following folic acid injury and Tanshinone IIA treatment, kidney specimens procured on day 28 were processed for Masson trichrome staining for collagens and peroxidase staining for fibronectin. Shown in Fig. 3a,b, folic acid injury resulted in conspicuous transition of AKI to CKD, marked by tubular atrophy and interstitial fibrosis that is characterized by substantial collagen and fibronectin accumulation in renal interstitium. This effect was significantly mitigated by both early and late treatments with Tanshinone IIA. The morphologic findings were further corroborated by immunoblot analysis of kidney homogenates for collagen I and fibronectin, followed by densitometry analysis of immunoblots (Fig. 3c–e), which demonstrated a greater beneficial efficacy of the early than the late Tanshinone IIA treatment. The seemingly superior efficacy of early *versus* late Tanshinone IIA treatment is unlikely attributable to the difference in treatment duration. In support of this, additional groups of mice were sacrificed 23 d after early Tanshinone IIA or vehicle treatment (i.e. 24 d after folic acid injury). Histologic signs of kidney destruction, AKI to CKD transition and renal fibrosis, as estimated by morphology based on Masson trichrome staining (Supplemental Figure 1a,b) or by immunoblot analysis of kidney specimens for collagen I and fibronectin (Supplemental Figure 1c~e), were all improved by early Tanshinone IIA treatment to a significantly greater extent when compared with late Tanshinone IIA treatment for the same period.

### Tanshinone IIA exerts a protective effect directly against the folic acid-elicited kidney damages

**Figure 4:**
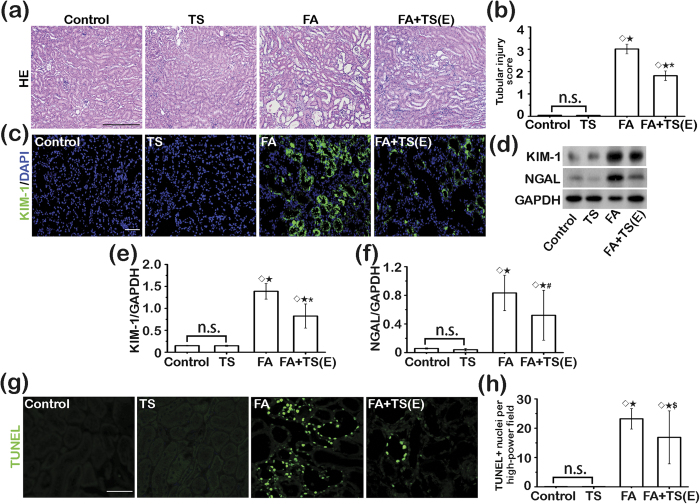
Early Tanshinone IIA treatment protects against folic acid-induced renal tubular damage. (**a**) Representative micrographs showing hematoxylin-eosin (HE) staining of kidney specimens procured on day 3. Scale bar = 500 μm. (**b**) Kidney tubular injury score assessed based on evaluation of HE staining. ^◊^*P* < 0.001 *vs* Control; ^★^*P* < 0.001 *vs* TS; ^*^P < 0.001 *vs* FA; n.s., not significant. (**c**) Representative micrographs showing immunofluorescent staining of kidney specimens procured on day 3 for kidney injury molecule-1(KIM-1). Sections were counterstained with 4′,6-diamidino-2-phenylindole (DAPI). Scale bar = 200 μm. (**d**) Representative immunoblots of KIM-1 and neutrophil gelatinase associated lipocalin (NGAL) in cortical kidney lysates. (**e**) and (**f**) Relative abundance of KIM-1 (**e**) and NGAL (**f**) in immunoblots expressed as densitometric ratios of KIM-1/GAPDH or NGAL/GAPDH; ^◊^*P* < 0.001 *vs* Control; ^★^*P* < 0.001 *vs* TS; ^*^P < 0.001 *vs* FA; ^#^*P* = 0.004 *vs* FA; n.s., not significant; (n = 9). (**g**) Representative micrographs showing TUNEL staining of kidney specimens procured on day 3. Scale bar = 200 μm. (**h**) Absolute counting of TUNEL-positive nuclei per high power field. ^◊^*P* < 0.001 *vs* Control; ^★^*P* < 0.001 *vs* TS; ^$^*P* = 0.009 *vs* FA; n.s., not significant; (n = 9).

One of the major characteristics of the post-AKI pathology is renal tubular epithelial destruction. To determine if Tanshinone IIA treatment directly affects renal tubular injury after folic acid exposure, kidney tissues were procured on day 3 and examined. Scoring of renal tubular injury based on H&E staining (Fig. 4a,b) revealed that early Tanshinone IIA therapy effectively ameliorated the folic acid-elicited renal tubular injuries. This finding was further validated by fluorescent immunohistochemistry staining of kidney sections for kidney injury molecule-1 (KIM-1, Fig. 4c) or immunoblot analysis of kidney homogenates for KIM-1 and NGAL (Fig. 4d~f), well established biomarkers for renal tubular injuries. Renal tubular death, marked by acute tubular necrosis and apoptosis, is a main feature of AKI. To evaluate the possible effect of Tanshinone IIA on renal tubular cell death, kidney specimens were prepared for terminal deoxynucleotidyl transferase dUTP nick end labeling (TUNEL) for apoptotic cells. Shown in Fig. 4g, folic acid insult elicited blatant apoptosis in the kidney with TUNEL staining largely distributed to tubular epithelia. This effect was considerably overridden by Tanshinone IIA treatment with a much stronger effect observed in the early treatment group. Morphologic findings were confirmed by absolute counting of TUNEL positive cells (Fig. 4h). Taken together, these data infer that Tanshinone IIA therapy conveys a direct protective effect on renal tubules.

### Tanshinone IIA inhibits early inflammatory response in folic acid-injured kidneys

**Figure 5:**
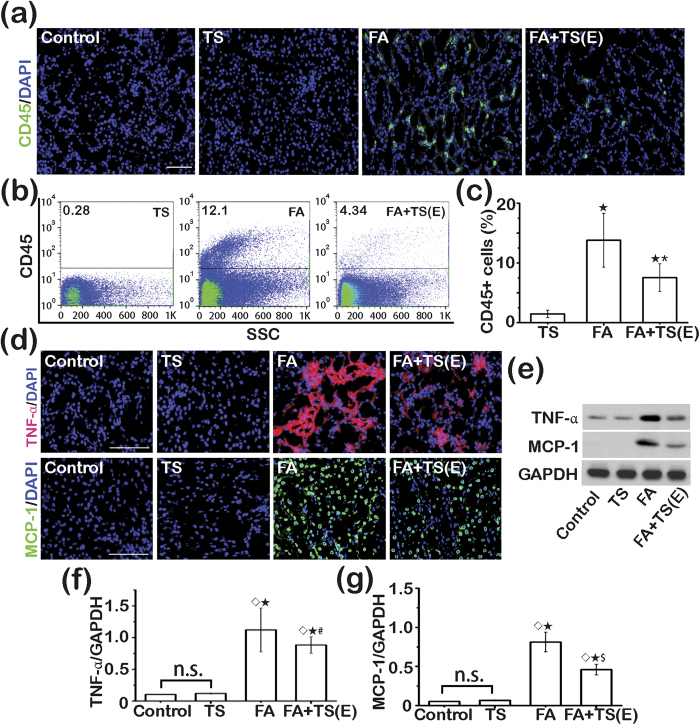
Early Tanshinone IIA treatment attenuates folic acid-induced kidney inflammation. (**a**) Representative micrographs showing immunofluorescent staining of kidney specimens procured on day 3 for CD45. Sections were counterstained with 4′,6-diamidino-2-phenylindole (DAPI). Scale bar = 200 μm. (**b**) Flow cytometry analysis of isolated single kidney cells for CD45^+^ cells. SSC, Side-scatter. (**c**) Quantitation of CD45^+^ cells in the kidney as measured by flow cytometry. ^★^*P* < 0.001 *vs* TS; ^*^P < 0.001 *vs* FA; (n = 9). (**d**) Representative micrographs showing immunofluorescent staining of kidney specimens procured on day 3 for tumor necrosis factor-α (TNF-α) or monocyte chemotactic protein-1 (MCP-1). Sections were counterstained with DAPI. Scale bar = 200 μm. (**e**) Representative immunoblots of TNF-α and MCP-1 in cortical kidney lysates. f and g, Relative abundance of TNF-α (**f**) and MCP-1 (**g**) in immunoblots expressed as densitometric ratios of TNF-α/GAPDH or MCP-1/GAPDH. ^◊^*P* < 0.001 *vs* Control; ^★^*P* < 0.001 *vs* TS; ^#^*P* = 0.008 *vs* FA; ^$^*P* = 0.003 *vs* FA; n.s., not significant; (n = 9).

To understand the mechanism responsible for the extra benefit conferred by early Tanshinone IIA treatment, kidney tissues were collected on day 3 and examined. As compared with kidney specimens from control or Tanshinone IIA alone treated animals, folic acid-injured kidneys exhibited florid infiltrations by inflammatory cells that were positive for leukocyte common antigen (CD45), as determined by fluorescent immunohistochemistry staining or by flow cytometry analysis of isolated single kidney cells (Fig. 5a~c). Early Tanshinone IIA treatment substantially reduced the number of CD45 positive cells in the kidney, denoting an anti-inflammatory activity. Local production of proinflammatory cytokines and chemokines is a prerequisite for recruitment of circulating leukocytes to periphery lesion sites. To further explore if the anti-inflammatory action of Tanshinone IIA possibly stems from a primary effect on the inflamed kidney, kidney specimens were prepared for fluorescent immunohistochemistry staining for tumor necrosis factor-α (TNF-α) and monocyte chemotactic protein-1 (MCP-1), which are cytokines crucial for inflammatory response and leukocyte infiltration. Shown in Fig. 5d, early Tanshinone IIA treatment drastically mitigated the folic acid-elicited renal expression of TNF-α, which was mainly distributed to renal tubules. The morphologic findings were further corroborated by confirmatory immunoblot analysis of kidney homogenates (Fig. 5e) followed by densitometric quantitation (Fig. 5f,g).

### Macrophage infiltration in folic acid-injured kidneys is attenuated by Tanshinone IIA therapy

**Figure 6:**
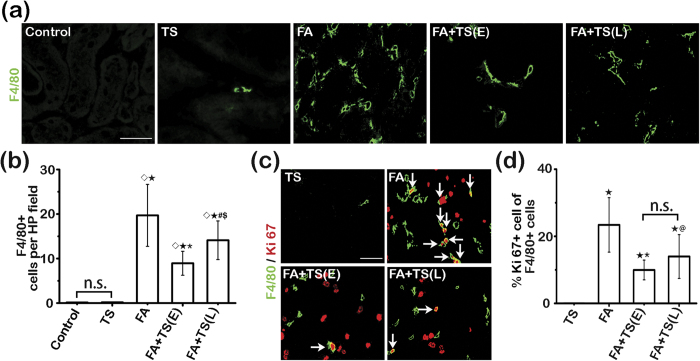
Tanshinone IIA mitigates macrophage accumulation in the folic acid injured kidney. (**a**) Representative micrographs showing immunofluorescent staining of kidney specimens procured on day 7 for F4/80. Scale bar = 200 μm. (**b**) Absolute counting of the number of F4/80-positive cell per high-power (HP) field. ^◊^*P* < 0.001 *vs* Control; ^★^*P* < 0.001 *vs* TS; ^*^P < 0.001 vs FA; ^#^*P* = 0.004 *vs* FA; ^$^*P* = 0.007 *vs* FA+TS(E); n.s., not significant; (**c**) Representative micrographs showing dual color immunofluorescent staining of kidney specimens procured on day 7 for F4/80 and Ki67. Scale bar = 100 μm. White arrows indicate the dually positive cells in the sections. (**d**) The percentage of F4/80^+^Ki67^+^ cells among all F4/80^+^ cells. ^★^*P* < 0.001 *vs* TS; ^*^P < 0.001 vs FA; ^@^*P* = 0.004 *vs* FA; n.s., not significant; (n = 9).

A growing body of evidence suggests that macrophages play a pivotal role in the development and progression of AKI. Kidney specimens collected on day 7 were examined for renal infiltration of macrophages by fluorescent immunohistochemistry staining for F4/80. Shown in Fig. 6a,b, F4/80 positive cells were barely detected in kidney tissues from control or Tanshinone IIA alone treated mice. Following folic acid injury, massive macrophage infiltration was noted in renal interstitium. Delayed treatment with Tanshinone IIA markedly attenuated macrophage accumulation in the folic acid-injured kidney with a greater effect observed in the early treatment group (Fig. 6b). Evidence indicates that macrophage proliferation may contribute to macrophage accumulation in injured tissues. To evaluate macrophage proliferation, dual color fluorescent immunohistochemistry staining for F4/80 and Ki 67 was conducted (Fig. 6c). The number of proliferative macrophages marked by cells positive for both F4/80 and Ki67 in the folic acid-injured kidney was evidently reduced after Tanshinone IIA treatment. These data indicate that Tanshinone IIA treatment impedes macrophage infiltration and proliferation in the folic acid injured kidneys.

### Tanshinone IIA treatment promotes M2 macrophage polarization in the folic acid injured kidney

**Figure 7:**
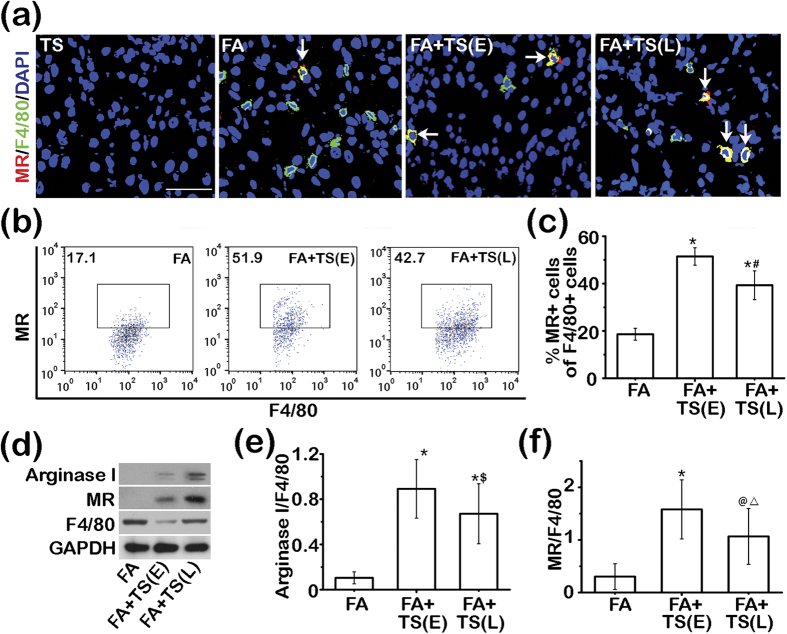
Tanshinone IIA promotes M2 macrophages polarization in the folic acid injured kidney. (**a**) Representative micrographs showing immunofluorescent staining of kidney specimens procured on day 7 for F4/80 and mannose receptor (MR). Sections were counterstained with 4′,6-diamidino-2-phenylindole (DAPI). Scale bar = 50 μm. White arrows indicate the dually positive cells in the sections. (**b**) Flow cytometry analysis of isolated single kidney cells for cells positive for MR and F4/80; cells were gated on F4/80^+^ macrophages. (**c**) The percentage of F4/80^+^MR^+^ cells among all F4/80^+^ cells as determined by flow cytometry. ^*^P < 0.001 *vs* FA; ^#^*P* < 0.001 *vs* FA+TS(E); (n = 9). (**d**) Representative immunoblots of Arginase I, MR, and F4/80 in cortical kidney lysates prepared on day 7. (**e**) and (**f**) Relative abundance of Arginase I (**e**) and MR (**f**) in immunoblots expressed as densitometric ratios of Arginase I/F4/80 or MR/F4/80. ^*^P < 0.001 *vs* FA; ^$^*P* = 0.041 *vs* FA+TS(E); ^@^*P* = 0.002 *vs* FA; ^△^*P* = 0.029 *vs* FA+TS(E); (n = 9).

Macrophages in diseased kidneys exist in different subtypes, such as M1 and M2, which play distinct or even opposite roles in renal injury and repair. Following folic acid injury, the majority of the infiltrating F4/80 positive macrophages were negative for mannose receptor (MR) on dual color fluorescent immunohistochemistry staining and flow cytometry analysis of isolated single kidney cell suspensions (Fig. 7a,b), consistent with an M1 phenotype. Tanshinone IIA treatment markedly increased the number of F4/80^+^MR^+^ M2 subtype of macrophages in the folic acid injured kidney with a greater effect observed in the early treatment group (Fig. 7a–c). In agreement, immunoblot analysis of kidney homogenates for M2 macrophage markers, like MR or arginase I, suggests that M2 polarization was considerably amplified after Tanshinone IIA treatment with a stronger effect observed in the early treatment group (Fig. 7d~f). These results imply that Tanshinone IIA treatment favors an M2-skewed macrophage polarization in the folic acid injured kidneys.

### Tanshinone IIA modulates glycogen synthase kinase (GSK) 3β-MAPK signaling pathway activity in the folic acid injured kidney

**Figure 8:**
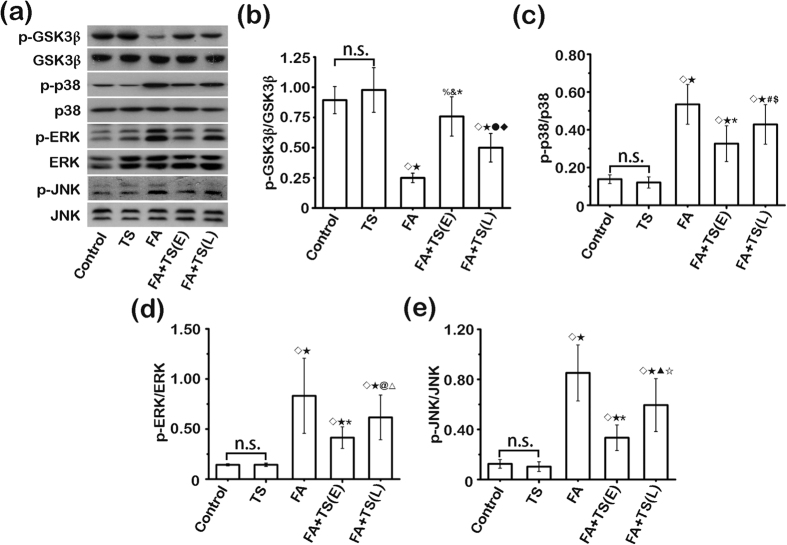
Tanshinone IIA mitigates GSK3β overactivity and hyperactivation of its downstream mitogen-activated protein kinases (MAPK) in folic acid-injured kidneys. (**a**) Representative immunoblots of GSK3β and phosphorylated GSK3β-Ser9(p-GSK3β); p38 and phosphorylated p38 (p-p38); extracellular regulated protein kinase (ERK) and phosphorylated ERK (p-ERK); c-Jun N-terminal kinase (JNK) and phosphorylated JNK (p-JNK) in cortical kidney lysates prepared from indicated animal groups on day 7. (**b**) Relative abundance of p-GSK3β in immunoblots expressed as densitometric ratios of p-GSK3β/GSK3β. ^◊^*P* < 0.01 *vs* Control; ^★^*P* < 0.01 *vs* TS; ^%^*P* = 0.041 *vs* Control; ^&^*P* = 0.027 *vs* TS; ^*^P < 0.001 *vs* FA; ^●^*P* = 0.004 *vs* FA; *P* = 0.014 *vs* FA+TS(E). (**c**) Relative abundance of p-p38 in immunoblots expressed as densitometric ratios of p-p38/p38; ^◊^*P* < 0.01 *vs* Control; ^★^*P* < 0.01 *vs* TS; ^*^P < 0.001 *vs* FA; ^#^*P* = 0.048 *vs* FA; ^$^*P* = 0.031 *vs* FA+TS(E). (**d**) Relative abundance of p-ERK in immunoblots expressed as densitometric ratios of p-ERK/ERK. ^◊^*P* < 0.01 *vs* Control; ^★^*P* < 0.01 *vs* TS; ^*^P < 0.001 *vs* FA; ^@^*P* = 0.029 *vs* FA; ^△^*P* = 0.033 *vs* FA+TS(E). (**e**) Relative abundance of p-JNK in immunoblots expressed as densitometric ratios of p-JNK/JNK. ^◊^*P* < 0.01 *vs* Control; ^★^*P* < 0.01 *vs* TS; ^*^P < 0.001 *vs* FA; ^▲^*P* = 0.007 *vs* FA; ^☆^*P* = 0.011 *vs* FA+TS(E); n.s., not significant; (n = 9).

Burgeoning evidence indicates that GSK3β is a point of convergence of multiple pathways involved in acute and chronic kidney injuries^38^. GSK3β has been known to dictate the activity of mitogen-activated protein kinases (MAPK)^39^,^40^,^41^, which are implicated in modulating renal inflammation and fibrogenesis^42^,^43^,^44^,^45^,^46^,^47^. Indeed, folic acid injury drastically induced GSK3β overactivity, reflected by the diminished inhibitory phosphorylation of GSK3β in the kidney, and triggered hyperphosphorylation and hyperactivation of p38 (Fig. 8a,c), extracellular regulated protein kinase (ERK, Fig. 8a,d) and c-Jun N-terminal kinase (JNK, Fig. 8a,e), as shown by immunoblot analysis of kidney homogenates. The augmented activity of GSK3β and diverse MAPK pathways was strikingly blunted following Tanshinone IIA treatment with a stronger effect detected in the early treatment group, concomitant with the anti-inflammatory and anti-fibrotic action of Tanshinone IIA observed above.

### Inhibition of GSK3β is a key signaling mechanism mediating the kidney protective effect of Tanshinone IIA

**Figure 9:**
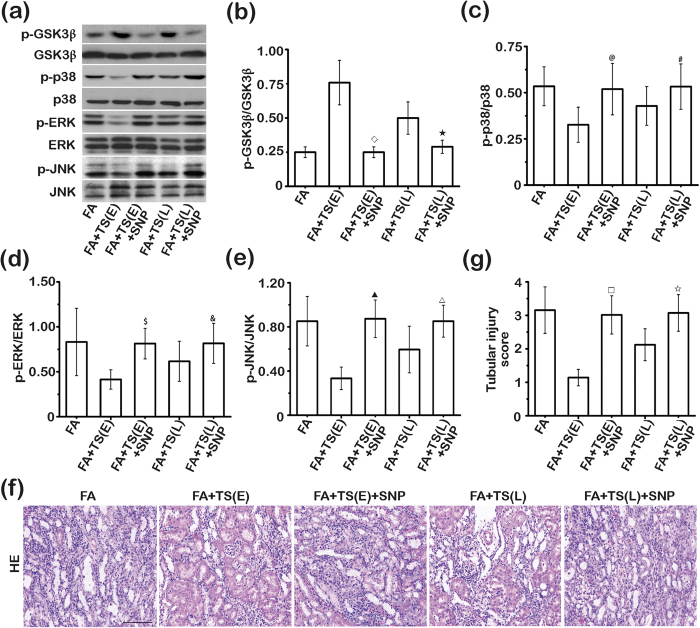
Inhibition of GSK3β is essential for the beneficial effect of Tanshinone IIA in folic acid-injured kidney. Mice received Tanshinone IIA therapy in the presence of daily treatment with vehicle or sodium nitroprusside (SNP, 200 μg/kg), a GSK3β activator, starting 1 day or 5 days after folic acid injury. Kidney specimens were procured day 7 after folic acid injury. (**a**) Representative immunoblots of GSK3β and phosphorylated GSK3β-Ser9(p-GSK3β); p38 and phosphorylated p38 (p-p38); extracellular regulated protein kinase (ERK) and phosphorylated ERK (p-ERK); c-Jun N-terminal kinase (JNK) and phosphorylated JNK (p-JNK) in cortical kidney lysates prepared on day 7. (**b**) Relative abundance of p-GSK3β in immunoblots expressed as densitometric ratios of p-GSK3β/GSK3β. ^◊^*P* < 0.001 *vs* FA+TS(E); ^★^*P* < 0.001 *vs* FA+TS(L). (**c**) Relative abundance of p-p38 in immunoblots expressed as densitometric ratios of p-p38/p38. ^@^*P* < 0.001 *vs* FA+TS(E); ^#^*P* = 0.027 *vs* FA+TS(L). (**d**) Relative abundance of p-ERK in immunoblots expressed as densitometric ratios of p-ERK/ERK. ^$^*P* < 0.001 *vs* FA+TS(E); ^&^*P* = 0.009 *vs* FA+TS(L). (**e**) Relative abundance of p-JNK in immunoblots expressed as densitometric ratios of p-JNK/JNK. ^▲^*P* < 0.001 *vs* FA+TS(E); ^△^*P* = 0.004 *vs* FA+TS(L). (**f**) Representative micrographs showing hematoxylin-eosin (HE) staining of kidney specimens procured on day 7. Scale bar = 200 μm. (**g**) Kidney tubular injury score assessed based on evaluation of H&E staining. *P* < 0.001 *vs* FA+TS(E); ^☆^*P* < 0.001 *vs* FA+TS(L); (n = 9).

GSK3β has been shown to play a crucial role in mediating kidney injury^38^. To determine if inhibition of GSK3β possibly represents a key mechanism conveying the beneficial activity of Tanshinone IIA after AKI, additional mice received Tanshinone IIA therapy in the presence of daily treatment with vehicle or sodium nitroprusside, a GSK3β activator^48^, starting 1 day or 5 days after folic acid injury. Kidney specimens were procured 7 days after folic acid injury for immunoblot analysis (Fig. 9a~e) and histologic evaluation (Fig. 9f,g). Shown in Fig. 9a~e, both early and late Tanshinone IIA therapy restored inhibitory phosphorylation of GSK3β and overrode hyperphosphorylation of the downstream MAPK kinases, including p38, ERK and JNK. These cellular signaling effects were markedly abrogated by simultaneous sodium nitroprusside treatment. In parallel, histological signs of kidney destruction (Fig. 9f), including vacuolization of proximal tubular epithelium, luminal ectasia, tubular epithelial simplification and atrophy, and inflammatory infiltration in tubulointerstitium were improved after early or late Tanshinone IIA therapy, as corroborated by semi-quantitative evaluation of tubular injury scores (Fig. 9g). The beneficial effect of Tanshinone IIA was markedly offset by sodium nitroprusside co-treatment, entailing that inhibition of GSK3β is essential for the kidney protective activity of Tanshinone IIA.

### Delayed administration of Tanshinone IIA attenuates kidney destruction following IRI

**Figure 10:**
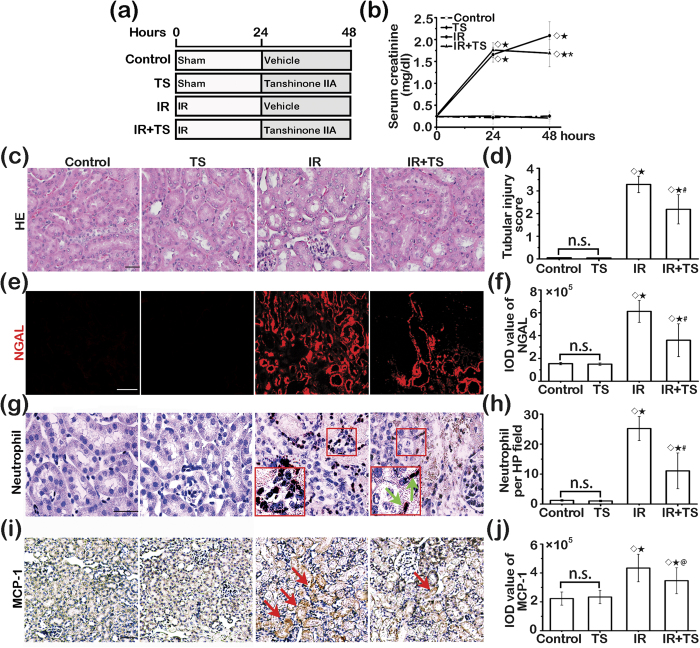
Tanshinone IIA attenuates ischemia-reperfusion injury induced renal dysfunction and kidney damage and inflammation. (**a**) Animal experimental design. Mice were randomized into four groups to receive bilateral kidney ischemia-reperfusion (IR) injury and treatment with vehicle or Tanshinone IIA (TS) given 24 hours after ischemia. (**b**) Time course changes of serum creatinine levels measured at 0, 24 and 48 hours. ^◊^*P* < 0.001 *vs* Control; ^★^*P* < 0.001 *vs* TS; ^*^P = 0.009 *vs* IR. (**c**) Representative micrographs showing hematoxylin-eosin (HE) staining of kidney specimens procured at 48 h. Scale bar = 200 μm. (**d**) Renal tubular injury score assessed based on evaluation of HE staining. ^◊^*P* < 0.001 *vs* Control; ^★^*P* < 0.001 *vs* TS; ^#^*P* = 0.008 *vs* IR. (**e**) Representative micrographs showing immunofluorescent staining of kidney specimens procured at 48 h for neutrophil gelatinase associated lipocalin (NGAL). Scale bar = 200 μm. (**f**) Semi-quantification of NGAL staining by morphometric analysis. ^◊^*P* < 0.001 *vs* Control; ^★^*P* < 0.001 *vs* TS; ^#^*P* = 0.011 *vs* IR. (**g**) Representative micrographs showing immunoperoxidase staining of kidney specimens procured at 48 h for neutrophils; Scale bar = 100 μm. (**h**) Absolute counting of the number of neutrophils positive for myeloperoxidase per high-power (HP) field. ^◊^*P* < 0.001 *vs* Control; ^★^*P* < 0.001 *vs* TS; ^#^*P* = 0.002 *vs* IR. (**i**) Representative micrographs showing immunoperoxidase staining of kidney specimens procured at 48 h for monocyte chmotactic protein-1 (MCP-1); Scale bar = 500 μm. (**j**) Semi-quantification of MCP-1 expression estimated by morphometric analysis. ^◊^*P* < 0.01 *vs* Control; ^★^*P* < 0.01 *vs* TS; ^@^*P* = 0.031 *vs* IR. n.s., not significant; (*n* = 6).

To further ascertain if the beneficial effect of delayed Tanshinone IIA therapy could be generalized to other types of AKI, an additional murine model of AKI induced by IRI was employed (Fig. 10a). Shown in Fig. 10b, Tanshinone IIA treatment given 24 hours after IRI markedly improved kidney function, reflected by the lowered levels of serum creatinine. This was accompanied with amelioration of renal histologic injuries, as revealed by scoring of tubular injury based on H&E staining and by fluorescent immunohistochemistry staining of NGAL in conjunction with morphometric analysis (Fig. 10c~f). Moreover, histologic signs of renal inflammation, including neutrophil infiltration and renal expression of MCP-1, in IR-injured kidneys were significantly attenuated after Tanshinone IIA therapy (Fig. 10g~j).

## Discussion

In this study, we found that delayed Tanshinone IIA therapy, commenced early or late after AKI, could effectively attenuate kidney injury elicited by folic acid or IRI and hinder AKI to CKD transition. This beneficial effect of Tanshinone IIA is likely associated with a direct protection against renal tubular damage and an anti-inflammatory activity. To the best of our knowledge, this study is the first to demonstrate the protective effect of delayed Tanshinone IIA therapy on established AKI and the ensuing transition to CKD.

The mechanism responsible for the protective effect of Tanshinone IIA on renal tubular damage is not clear, but our data suggest that it might be associated with a direct anti-apoptotic action. However, the effect of Tanshinone IIA on cellular apoptosis has been highly controversial with both proapoptotic and antiapoptotic activities reported before. For instance, in cardiomyocytes injured with doxorubicin or angiotensin II, Tanshinone IIA was able to confer a potent anti-apoptotic effect^49^,^50^. Similarly, in cultured endothelial cells, Tanshinone IIA was capable of counteracting the proapoptotic effect of hydrogen peroxide^51^. In contrast, Tanshinone IIA has been shown to repress cell growth through inducing apoptosis in multiple cancer cells, including leukemia cells, osteosarcoma cells, and prostate cancer cells^52^,^53^,^54^. This disparity might be due to distinct responses of different cell types, but is more likely associated with difference in Tanshinone IIA doses. Indeed, the doses of Tanshinone IIA applied in studies showing proapoptotic activities^53^ were almost 10 times higher than those used in studies demonstrating cytoprotective effects^49^. This seemingly bimodal effect of Tanshinone IIA has been leveraged to kill cancer cells at high doses, but to protect parenchymal cells at much lower doses. The dose of Tanshinone IIA used in this study was very low (50 mg/kg/d) and had been reported by other studies to be safe^23^,^55^. No noticeable adverse effect was noted in our animals, according to the observation of body weight, daily behavior and activity and to the analysis of other organ specimens. In this study, Tanshinone IIA treatment lessened renal tubular damage and apoptosis following AKI. Whether this effect was exerted *via* a renal tubular cell autonomous mechanism warrants in-depth exploration.

In contrast to the arguable effect on apoptosis, Tanshinone IIA has been reproducibly demonstrated to possess a potent anti-inflammatory activity in animal models of diverse diseases, including chronic kidney disease, ischemic brain injury, endotoxin-associated acute lung injury, ischemic heart disease and atherosclerosis^19^,^23^,^55^,^56^. However, it is largely unknown whether Tanshinone IIA is also beneficial in AKI or whether Tanshinone IIA is able to alter inflammatory injuries in AKI. Inflammation *per se* is involved in the pathogenesis of AKI and plays a key role in the following transition to CKD. Inflammatory cell infiltration, a hallmark of inflammatory response, is not only closely associated with the severity of AKI, but also a crucial determinant of the long-term outcome of the injured kidney. Data from animal experiments showed that neutrophil infiltration can be detected as early as just few hours after kidney injury. Although the role of neutrophil infiltration in AKI remains to be defined, recent evidence suggests that blocking the recruitment of neutrophil into the kidney can effectively protect the IRI incited kidney injury^57^. In consistency, in the present study, we found that delayed Tanshinone IIA treatment after initial insult could significantly decrease the numbers of leukocytes in the folic acid injured kidneys and the amount of neutrophils in IR injured kidneys, in parallel with amelioration of kidney dysfunction and renal histologic injury. This finding is in accordance with some previous studies demonstrating that Tanshinone IIA could impede neutrophils infiltration into lipopolysaccharide (LPS)-injured lung or the brain following ischemia injury^23^,^29^. In conjunction with previous findings, it seems that Tanshinone IIA may ameliorate established AKI in part by suppressing inflammatory infiltration at the acute phase.

Macrophage is a key player in the development and progression of AKI^58^. They usually infiltrate into the injured kidney several days after the initial insult. Emerging data prove that the numbers of infiltrating macrophages at the early phase of injury is associated with the severity of AKI^59^. In consistency, early depletion of macrophages significantly attenuated the renal injury in various models of AKI. In our study, both early and late treatments with Tanshinone IIA after AKI could significantly reduce the accumulation of macrophage in folic acid injured kidney. The underlying mechanism is not clear. One possibility is that Tanshinone IIA may be able to inhibit *in situ* macrophage proliferation, as shown by our results. Another possibility could be that Tanshinone IIA directly hampers macrophage recruitment into the kidney. In support of this, Tanshinone IIA treatment inhibited the expression of chemokines like MCP-1 in renal parenchyma after folic acid injury or IRI^60^. Different macrophage subtypes are known to play distinct or even opposite roles in tissue injury and repair^59^,^61^. It is well known that M1 macrophages possess pro-inflammatory activities by releasing cytokines that damage surrounding tissue, whereas M2 macrophages exert anti-inflammatory effects by releasing cytokines that promote tissue recovery^61^,^62^,^63^. In the present study, Tanshinone IIA was found to favor an M2-skewed macrophage polarization in folic acid injured kidneys, suggestive of a proreparative effect. This finding is consistent with a previous study showing that Tanshinone IIA could inhibit the expression of inducible nitric oxide synthase in LPS-stimulated macrophages *in vitro*^64^,^65^. However, whether the effect of Tanshinone IIA on macrophage polarization in the injured kidney is attributable to a direct effect on macrophages *per se* or secondary to the improved local tissue microenvironments merits further investigation.

The molecular basis of progression of AKI is complex. Accumulating evidence points to GSK3β as a point of convergence for a number of nephropathic pathways downstream diverse renal injurious signals. GSK3β has been known to dictate the activity of MAPK kinases^39^,^40^,^41^, including p38, ERK and JNK, which have been implicated in renal inflammation and fibrogenesis in progressive AKI^38^. MAPKs are implicated in NF-κB activation and production of proinflammatory cytokines and chemokines in the kidney upon injury^42^,^43^,^44^. In addition, MAPKs play a critical role in AKI to CKD transition or renal fibrogenesis, characterized by tubular atrophy and interstitial fibrogenesis^45^,^46^,^47^. Furthermore, activation of MAPKs is associated with renal fibroblast proliferation and activation^46^, a hallmark of kidney fibrogenesis. In our study, Tanshinone IIA treatment was able to effectively mitigate GSK3β overactivity and subsequent hyperactivation of MAPK pathways in the injured kidney. Conversely, sodium nitroprusside, a GSK3b activator^48^, offset the effect of Tanshinone IIA on the GSK3β/MAPK kinases signaling pathways and abolished its renoprotective activity, suggesting that the fundamental mechanism responsible for the beneficial effect of Tanshinone IIA in AKI may lies in its regulation of the signaling activity of GSK3β/MAPK kinases pathway.

In aggregate, our study demonstrated that delayed treatment with Tanshinone IIA in established AKI can effectively ameliorate progressive kidney destruction through protecting against kidney damage and inhibiting inflammation. Our findings suggest that Tanshinone IIA represents a novel and pragmatic therapeutic strategy that rescues the kidney from AKI via a multi-pronged mechanism. Future clinical trials are certainly merited to examine its safety and effectiveness in AKI patients.

## Materials and Methods

The Animal Care and Use Committee at Nanjing University approved the animal studies, which conformed to the United States Department of Agriculture regulations and the National Institutes of Health guidelines for humane care and use of laboratory animals. C57BL/6 male mice aged 6 to 8 weeks and weighing 20 to 22 grams were obtained from Nanjing University Model Animal Research Center and used for this study. Six to nine mice were randomly assigned to each group for each observed time point.

### Animal Experimental Design

#### Murine Models of folic acid-induced AKI

Mice were randomized to receive an intraperitoneal injection of folic acid (250 mg/kg, Sigma-Aldrich, St. Louis, MO, USA) dissolved in 0.3 mM sodium bicarbonate (FA) or equal volume of vehicle (0.3 mM sodium bicarbonate) on day 0. Starting day 1, mice were randomly assigned to receive daily Tanshinone IIA (50 mg/kg/d, Jiangsu Carefree Group Co., Nanjing, China) in corn oil [TS(E)] according to the doses safely used in animal models in published studies^22^,^23^,^24^,^25^,^26^,^27^,^28^,^29^,^30^,^31^,^32^,^33^,^34^,^35^,^36^,^37^ or an equal volume of corn oil by gavage. For late Tanshinone IIA treatment [TS(L)] treatment, some folic acid-injured mice were fed daily with Tanshinone IIA by gavage starting day 5. Some Tanshinone IIA-treated mice were co-treated intraperitoneally with 200 μg/kg/d sodium nitroprusside (Sigma) or its vehicle daily. Animals were euthanized on post injury days 3, 7 and 28, followed by organ harvest for further investigation (Fig. 1a). Additional mice were killed on day 24 after injury. On indicated days, blood and urine samples were collected.

#### Murine Models of Renal IRI

Mice were anesthetized by ketamine (75 mg/kg) and dexdomitor (50 mg/kg). A flank incision was made on both left and right sides. The bilateral renal arteries and veins were isolated from the surrounding tissue by blunt dissection and then occluded with a nontraumatic vascular clamp for 30 min at 37 °C. In the sham group, renal pedicles were isolated but no clamps were applied. At the end of the ischemic period, the vascular clamps were removed and the kidneys were observed for 5 minutes to document reflow. At 24 h after the surgery, all mice started to receive daily Tanshinone IIA (50 mg/kg) in corn oil or an equal volume of corn oil by gavage. Blood and urine samples were collected at 24 and 48 hours and all animals were euthanized 48 h after IRI (Fig. 10a).

#### Blood and urine biochemistry analyses

Serum creatinine levels were measured using commercial assay kits according to the manufacturer’s instructions (K625–100, Biovision, Milpitas, CA, USA). Urinary NGAL concentration was determined by a commercially available ELISA test kit (Abcam, Cambridge, MA, USA).

#### Tissue preparation and single kidney cell isolation

Mice were sacrificed and perfused with ice-cold PBS via the left ventricle for 2 minutes. The right kidney was prepared for histologic examination. The left kidney was collected and enzymatically digested for flow cytometry analysis. In brief, kidneys were minced and incubated with collagenase IV (10 mg/mL, Sigma-Aldrich) and DNase I (200 U/mL, Sigma) in Hanks buffer for 20 minutes at 37 °C using a gentleMACS Dissociator (Miltenyi Biotec Inc, San Diego, CA, USA). Digestion was stopped by addition of fetal bovine serum. Samples of tissue suspensions were passed through a 100-μm cell strainer (Thermo Fisher Scientific, Waltham, MA, USA) and centrifuged at 300 *g* for 5 minutes at 4 °C to collect the pellet, which was then incubated with ACK Lysing Buffer (0.15 M NH_4_Cl, 10 mM KHCO_3_, and 0.1 mM Na_2_EDTA) to remove red blood cells. The samples were centrifuged again and pellet was then resuspended with PBS containing 1% BSA and 0.1% sodium azide (Sigma-Aldrich) and passed through a 40-μm cell strainer. The isolated single kidney cells were processed for flow cytometry.

#### Flow cytometry

The isolated single kidney cells were analyzed by flow cytometry (FACSArray; BD Biosciences, San Jose, CA USA) for CD45, F4/80 and MR expression. The following antibodies conjugated with fluorochromes were used: PE-labeled anti-CD45 (BD Biosciences), PE-labeled anti-F4/80 (eBioscience, San Diego, CA, USA), FITC-labeled anti-MR (BD Biosciences). Antibodies and their isotype controls were used according to the manufacturer’s recommendations. Data were collected on a FACSArray flow cytometer (BD Biosciences) and analyzed using FlowJo software (TreeStar, Ashland, OR, USA).

#### Renal morphology assessment

Formalin fixed paraffin embedded kidney specimens were prepared into 3-μm–thick sections. Sections were subjected to H&E staining to determine gross histological change or Masson trichrome staining to evaluate collagen deposition. One observer performed semi-quantitative morphometric analysis in a blinded manner. Tubular injury scores and interstitial cell infiltration scores were accessed based on HE staining and fibrosis scores were assessed based on Masson trichrome staining by using semi-quantitative measurements according to the proportion relative to the total section area and classified as follows: 0 (nil), 1 (<25%), 2 (25–50%), 3 (50–75%), and 4 (>75% of tubulointerstitial fields). Each score was determined by evaluating five random fields per section and three sections per kidney.

#### Peroxidase immunohistochemistry

Paraffin sections were deparaffinized and rehydrated. After antigen retrieval, sections were incubated overnight with antibodies against fibronectin (ab2413, Abcam) at 1:100 dilution, MCP-1 (ab25124, Abcam) at 1:200 dilution, or anti-neutrophil antibody (ab2557, Abcam) at 1:200 dilution at 4 °C. The ImmPRESS peroxidase polymer detection kit (Vector Laboratories, Burlingame, CA, USA) was used to conjugate secondary antibody for 30 minutes at 37 °C. Slides were finally developed with DAB substrate (Vector Laboratories). Computerized morphometric analysis of MCP-1staining was performed by using the Image Pro-Plus software (Media-Cybernetics, Silver Spring, MD). The final value was determined based on measuring five random fields per section and three sections per kidney.

#### Fluorescence immunohistochemistry staining

Frozen sections were fixed and incubated overnight at 4 °C with antibodies against CD45 (sc-25590, Santa Cruz Biotechnology, Santa Cruz, CA, USA) at 1:150 dilution, F4/80 (ab6640, Abcam) at 1:200 dilution, MR (AF2535, R&D Systems, Minneapolis, MN, USA) at 1:150 dilution, MCP-1 (ab25124, Abcam) at 1:150 dilution, NGAL (ab63929, Abcam) at 1:200 dilution, TNF-α (ab6671, Abcam) at 1:250 dilution, Ki-67 (AB9260, EMD Millipore, Darmstadt, Germany) at 1:400 dilution and KIM-1 (AF1817, R&D Systems) at 1:150 dilution. FITC-conjugated secondary antibodies (Invitrogen, CA, USA) against various species were incubated for 1 hour at room temperature. Finally, sections were counterstained with 4′,6-diamidino-2-phenylindole (H-1200, Vector Laboratories), and visualized using a fluorescence microscope. As a negative control, the primary antibodies were replaced by preimmune IgG from the same species; no staining occurred. The positively stained cells in the sections were counted in five random fields per section and three sections per kidney. The staining of NGAL was quantitated by computerized morphometric analysis of five random fields per section and three sections per kidney using Image Pro-Plus software (Media-Cybernetics, Silver Spring, MD).

#### Western Blotting Analysis

Cortical kidney lysates with equal amounts of total protein (50 μg/ml) were fractionated by 7.5–15% SDS-polyacrylamide gels under reducing conditions and analyzed by immunoblot. The antibodies against fibronectin, collagen I (ab34710, Abcam), MR, arginase I (ab64693, Abcam), NGAL, KIM-1, TNF-α, p38 (sc-535, Santa Cruz Biotechnology), MCP-1, phosphorylated-p38 (p-p38, 9211, Cell Signaling Technology, Danvers, MA, USA), ERK1/2 (sc-292838, Santa Cruz Biotechnology), GSK3β (sc-53931, Santa Cruz Biotechnology), phosphorylated GSK 3β-Ser 9 (p-GSK3β-Ser 9, sc-11757, Santa Cruz Biotechnology), phosphorylated ERK (p-ERK 1/2, 9101, Cell Signaling Technology), JNK (sc-572, Santa Cruz Biotechnology), phosphorylated JNK (p-JNK, ab124956, Abcam) and GAPDH (sc-48166, Santa Cruz Biotechnology) were used as primary antibodies.

#### TUNEL staining

TUNEL staining was performed on frozen sections with a DeadEnd Fluorometric TUNEL System kit (DeadEnd Fluorometric TUNEL System kit (G3250, Promega, USA) according to the manufacturer’s instructions. The number of TUNEL-positive nuclei per field was evaluated in five fields per section and three sections per kidney.

#### Statistical Analysis

Data are expressed as mean ± standard error. One-way analysis of variance was applied for multiple comparisons of values, followed by the post hoc Student-Newman-Keuls test. A value of p < 0.05 was considered statistically significant, and all tests were two-tailed. All statistical analyses were performed with the SPSS software (version 17.0 for Windows: SPSS Institute, Chicago, IL, USA).

## Additional Information

**How to cite this article**: Jiang, C. *et al*. Rescue therapy with Tanshinone IIA hinders transition of acute kidney injury to chronic kidney disease *via* targeting GSK3β. *Sci. Rep.* **6**, 36698; doi: 10.1038/srep36698 (2016).

**Publisher’s note:** Springer Nature remains neutral with regard to jurisdictional claims in published maps and institutional affiliations.

## Supplementary Material

Supplementary Information
