# Supplementary material for: Rescue therapy with Tanshinone IIA hinders transition of acute kidney injury to chronic kidney disease via targeting GSK3β
Source: Sci Rep. 2016 Nov 18;6:36698. doi: 10.1038/srep36698 (PMC5114614; doi:10.1038/srep36698)
Supplement: Supplementary Information [file srep36698-s1.pdf]

Rescue therapy with Tanshinone IIA hinders transition of acute kidney injury to chronic kidney disease via targeting GSK3β

Chunming Jiang[1, 4], Wei Zhu[1], Xiang Yan[2], Qiuyuan Shao[1], Biao Xu[3], Miao Zhang[1], Rujun Gong[4]

[1]*Department of Nephrology,* [2]*Department of Urology and* [3]*Department of Cardiology, Affiliated Nanjing Drum Tower Hospital, Nanjing University School of Medicine, Nanjing, China;* [4]*Division of Kidney Disease and Hypertension, Department of Medicine, Rhode Island Hospital, Brown University School of Medicine, Providence, Rhode Island, USA.*

Supplementary Figure S1

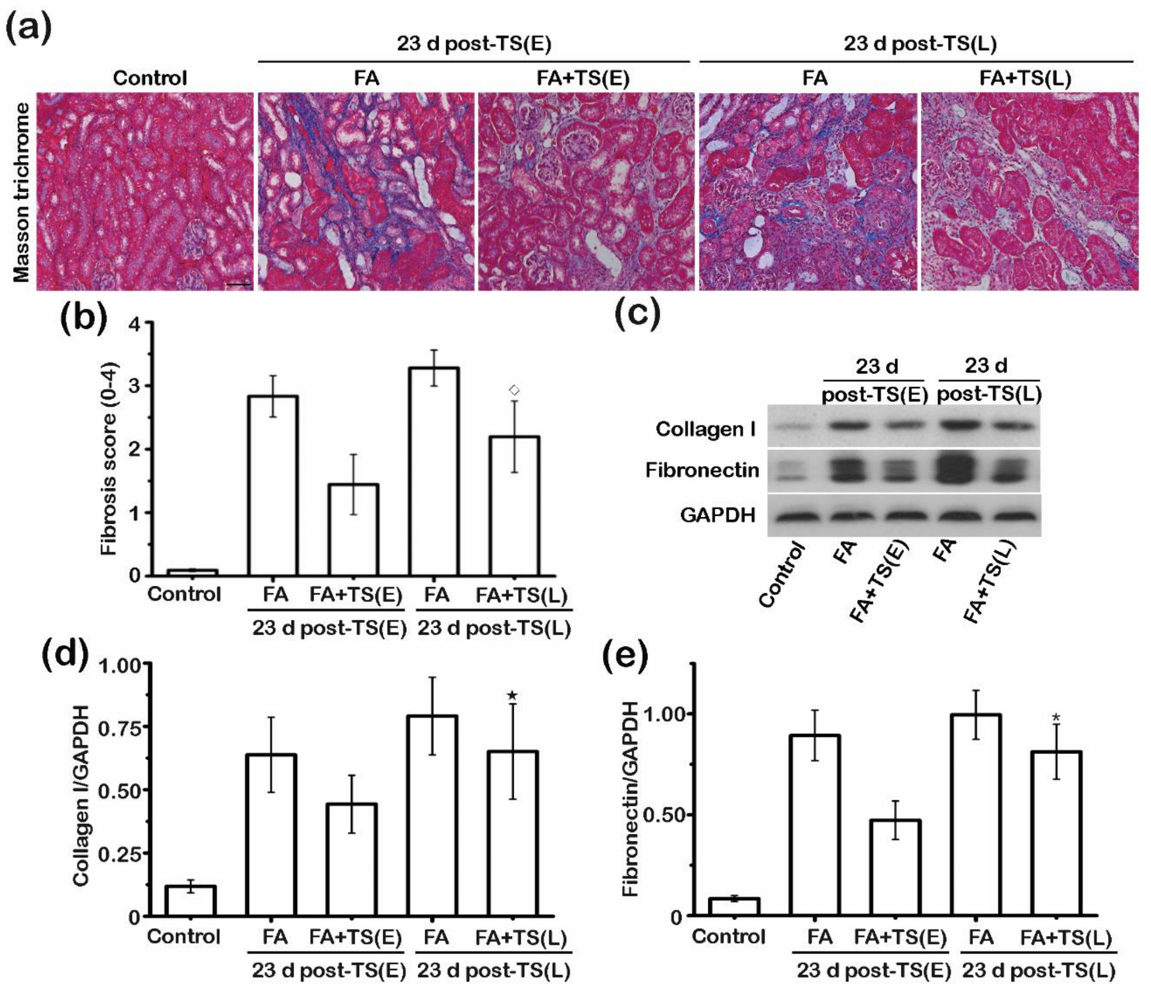

Supplementary Figure S1. The superior therapeutic efficacy of early *versus* late Tanshinone IIA treatment is unlikely attributable to the difference in treatment duration. a, Representative micrographs showing Masson trichrome staining of kidney specimens procured on day 23 after either early or late Tanshinone IIA administrstion; Scale bar = 200 μm. b, Kidney fibrosis score assessed based on evaluation of Masson trichrome staining. $^{\diamond}$*P*=0.026 vs FA+TS(E). c, Representative immunoblots of collagen I and fibronectin in cortical kidney lysates. d and e, Relative abundance of collagen I (d) and fibronectin (e) in immunoblots expressed as densitometric ratios of collagen I/GAPDH or fibronectin/GAPDH. **P*=0.033 *vs* FA+TS(E); **P*=0.017 *vs* FA+TS(E); (*n*=9).
